# Predictors of Atrial Fibrillation Recurrence in Hyperthyroid and Euthyroid Patients

**DOI:** 10.5935/abc.20160085

**Published:** 2016-06

**Authors:** Levent Cerit

**Affiliations:** Department of Cardiology - Near East University Hospital, Nicosia, Cyprus

**To the Editor,**

I have read with great interest the article entitled "Predictors of Atrial Fibrillation Recurrence in Hyperthyroid and Euthyroid Patients" by Gürdogan et al.,^1^ recently published in *Arquivos Brasileiros de Cardiologia.* The investigators reported that rates of atrial fibrillation (AF) recurrence were similar in hyperthyroid and euthyroid patients and that the duration of AF was the only predictor of AF recurrence in both.

Hyperthyroidism is a well-known risk factor for paroxysmal and permanent AF. Marrakchi et al.^2^ have reported that a low serum thyroid-stimulating hormone (TSH) level is an independent risk factor for AF. All other factors predisposing to AF were mentioned and discussed in that article.

Additionally, Demir et al.^3^ have found a strong relationship between vitamin D deficiency and nonvalvular AF. Serum vitamin D levels correlated with high sensitive C-reactive protein levels and left atrial diameter, and were significantly associated with AF in Chinese patients with nonvalvular persistent AF.^4^ Hanafy et al.^5^ have revealed the direct electromechanical effects of vitamin D administration on the left atrium and found that vitamin D could effectively prevent and terminate AF.

In the light of this knowledge, Gürdogan et al.^1^ should have reported the vitamin D levels of the patients in their study and discussed the association between the levels of this vitamin and AF recurrence.
