# Supplementary material for: Predictors of Atrial Fibrillation Recurrence in Hyperthyroid and Euthyroid Patients
Source: Arq Bras Cardiol. 2016 Jun;106(6):546–7. doi: 10.5935/abc.20160085 (PMC4940156; doi:10.5935/abc.20160085)
Supplement: Related texts

# Reply

**Authors:** Hasan ARI

We are pleased that Dr. Cerit showed great interest in our article entitled ''Predictors of Atrial Fibrillation Recurrence in Hyperthyroid and Euthyroid Patients'' published in *Arquivos Brasileiros de Cardiologia.*^1^

Recent studies have found that vitamin D deficiency is related to nonvalvular AF.^2,3^ However, the relationship between this deficiency and nonvalvular AF is not dependent on the occurrence of thyroid disorder. In the study published by Demir et al.,^2^ the TSH levels were normal in all AF groups. In addition, thyroid dysfunction was an exclusion criterion in the studies by both Demir et al.^2^ and Chen et al.^3^ We did not evaluate the vitamin D levels in our study's participants, which we can add as a limitation of our research. However, the participants in our study did not report any symptom or treatment of vitamin D deficiency. After thyroid surgery, in particular, patients may have vitamin D deficiency and hypothyroidism, but none of the patients in our study had prior thyroid surgery.

Considering the above, large-scale trials are still necessary to evaluate the relationship between vitamin D levels, thyroid function, and AF. We thank Dr. Cerit for this great contribution to our work.

Dr. Hasan ARI

## References

[1] Gürdogan M, Ari H, Tenekecioglu E, Ari S, Bozat T, Koca V (2016). Predictors of atrial fibrillation recurrence in hyperthyroid
and euthyroid patients. Arq Bras Cardiol.

[2] Demir M, Uyan U, Melek M (2014). The effects of vitamin D deficiency on atrial
fibrillation. Clin Appl Thromb Hemost.

[3] Chen WR, Liu ZY, Shi Y, Yin W, Wang H, Sha Y (2014). Relation of low vitamin D to nonvalvular persistent atrial
fibrillation in Chinese patients. Ann Noninvasive Electrocardiol.
